# Conformal High-Aspect-Ratio Solid Electrolyte Thin Films for Li-Ion Batteries by Atomic Layer Deposition

**DOI:** 10.1021/acsaelm.3c01565

**Published:** 2024-03-08

**Authors:** Milad Madadi, Mari Heikkinen, Anish Philip, Maarit Karppinen

**Affiliations:** ‡ Department of Chemistry and Materials Science, Aalto University, Espoo FI-00076, Finland; § Chipmetrics Ltd., Yliopistokatu 7, Joensuu FI-80130, Finland

**Keywords:** atomic layer deposition, thin film, conformality, solid electrolyte, LiPON, Li-ion battery

## Abstract

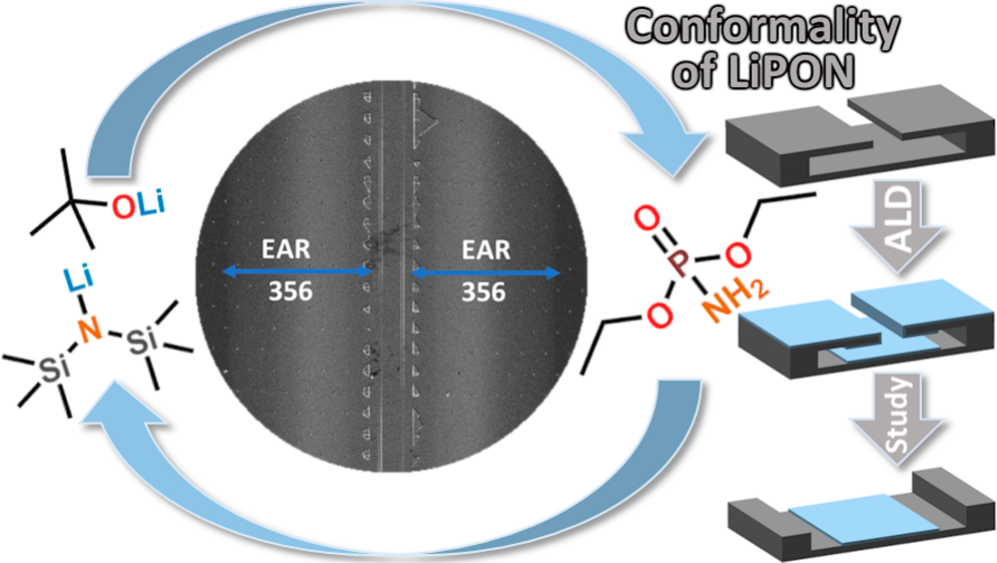

Lithium phosphorus oxynitride (LiPON) is a state-of-the-art solid electrolyte material for thin-film microbatteries. These applications require conformal thin films on challenging 3D surface structures, and among the advanced thin-film deposition techniques, atomic layer deposition (ALD) is believed to stand out in terms of producing appreciably conformal thin films. Here we quantify the conformality (i.e., the evenness of deposition) of thin ALD-grown LiPON films using lateral high-aspect-ratio test structures. Two different lithium precursors, lithium *tert*-butoxide (LiO^*t*^Bu) and lithium bis(trimethylsilyl)amide (Li-HMDS), were investigated in combination with diethyl phosphoramidate as the source of oxygen, phosphorus, and nitrogen. The results indicate that the film growth proceeded significantly deeper into the 3D cavities for the films grown from LiO^*t*^Bu, while the Li-HMDS-based films grew more evenly initially, right after the cavity entrances. These observations can be explained by differences in the precursor diffusion and reactivity. The results open possibilities for the use of LiPON as a solid electrolyte in batteries with high-surface-area electrodes. This could enable faster charging and discharging as well as the use of thin-film technology in fabricating thin-film electrodes of meaningful charge capacity.

The demand for mobile energy storage has increased with the rapid proliferation of miniaturized off-grid electronic devices. Li-based all-solid-state thin-film microbatteries (TFBs) provide a solution to the challenges that the conventional liquid-electrolyte-based Li-ion batteries and the currently increasingly investigated bulk solid-state batteries face in the size development of power sources.^1,2^ However, the significantly lower power and energy densities of TFBs compared to the state-of-the-art Li-ion batteries have so far restricted them from emerging as consumer products. The performance of TFBs depends on the surface area of their active battery materials.^3^ This can be increased, without greatly increasing the dimensions of the batteries, by 3D-structuring of the active materials.

For use in TFBs, many solid electrolyte material candidates have been developed. At the top of this list is lithium phosphorus oxynitride, LiP_*x*_O_*y*_N_*z*_ (LiPON),^4−6^ due to its many advantages such as its relatively high ionic conductivity, good electrochemical stability against Li metal, and low enough electronic conductivity. In addition to the aforementioned properties, thin LiPON films are transparent and can be deposited as part of flexible TFBs.^7,8^ So far, a variety of advanced gas-phase thin-film deposition techniques have been adopted for the growth of LiPON films on planar substrates, including sputtering,^9−11^ pulsed laser deposition (PLD),^12^ metal–organic chemical vapor deposition (CVD),^13^ and atomic layer deposition (ALD).^8,14−24^ The line-of-sight operation mode in both sputtering^25,26^ and PLD makes them unsuitable for complex 3D geometries. On the other hand, CVD techniques—especially ALD, which is based on self-saturating surface reactions—are anticipated to yield uniform and conformal coatings on complex 3D structures with large ARs.^27^ Indeed, the unique sequential precursor pulsing mode in ALD forms the basis for the desired atomic-level film-thickness control and large-area homogeneity and conformality (evenness of growth) of the films. These are the game-changing characteristics urgently looked for in next-generation 3D-structured solid-electrolyte layers in TFBs.^1,4,25−29^ Additionally, such conformal LiPON coatings could also serve as optimal interface layers in bulk solid-state battery configurations to improve the solid/solid interfacial contact between the electrode and electrolyte.^1^

There are two major ALD approaches to depositing high-quality LiPON thin films: (i) thermal ALD processes based on diethyl phosphoramidate (DEPA), which contains the two important elements, P and N, bonded together and (ii) plasma-assisted ALD processes based on several smaller N-, P-, and O-containing precursor molecules and either N_2_ or O_2_ plasma; the pioneering works in both cases were reported in 2015.^14,15^ Since then, more LiPON ALD studies have been published (Table 1). In these depositions, two different Li precursors have been employed: lithium *tert*-butoxide (LiO^*t*^Bu) and lithium bis(trimethylsilyl)amide (also known as lithium hexamethyldisilazide, Li-HMDS). In some of these studies, the conformality of growth was addressed but only using test structures with an aspect ratio (AR) of 25 at a maximum.^14,17,24^

**Table 1:** Previously Published ALD LiPON Processes: Precursors and *T*_dep_ Values Used, the Optimized GPC Values Obtained (If Given), and the Intended Applications^a^

year	precursors	*T*_dep_ (°C)	GPC (Å/cycle)	use	ref
2015	Li-HMDS/LiO^*t*^Bu, DEPA	270–310	0.6–0.7	SE	(14)
2015	LiO^*t*^Bu, H_2_O, PO(OMe)_3_, ^P^N_2_	250	1.05	SE	(15)
2016	LiO^*t*^Bu, NH_3_, P(NMe_2_)_3_, O_2_	350–500	0.72	SE	(16)
2017	LiO^*t*^Bu, DEPA	200–300	0.9	SE	(17)
2017	LiO^*t*^Bu, H_2_O, PO(OMe)_3_, ^P^N_2_	250	0.8	SE	(18)
2018	Li-HMDS, DEPA	300	0.6	TFB	(8)
2019	LiO^*t*^Bu, H_2_O, PO(OMe)_3_, ^P^N_2_	200–275		SE	(24)
2020	LiO^*t*^Bu, DEPA	200–300	0.9	SSB	(19)
2022	Li-HMDS, DEPA	300		high-*k*	(20)
2022	Li-HMDS, DEPA	300–330		Inh	(21)
2023	LiO^*t*^Bu, DEPA	350		Cap	(22)
2023	LiO^*t*^Bu, H_2_O, PO(OMe)_3_, ^P^N_2_	250		Cap	(22)

a SE = solid electrolyte; TFB = thin-film battery; SSB = solid-state battery; high-*k* = high-*k* dielectric; Inh = surface inhibition; Cap = capacitor.

In the present study, we have for the first time used lateral high-aspect-ratio (LHAR) test structures (AR > 10000) with submicron 3D features^30^ to investigate how deep and evenly LiPON films could grow in such LHAR structures. In previous works, similar test structures were utilized for other ALD thin-film materials to quantify the film penetration into the 3D trenches.^27,31^ Our data are for two different thermal ALD LiPON processes, based on LiO^*t*^Bu and Li-HMDS precursors, in combination with DEPA as the source of P, O, and N (Figure 1). We will demonstrate that the films grown from LiO^*t*^Bu penetrate deeper into the high-aspect-ratio structures. It should be emphasized that this comparative study was possible because both processes were carried out in the same commercial ALD reactor and with optimal deposition parameters for each process.

**Figure 1:**
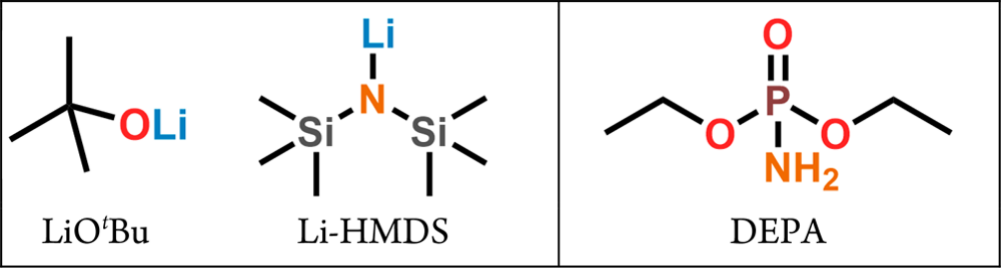
Molecular structures of the precursors used in this study.

The detailed experimental parameters are found in the Supporting Information (SI; Experimental Details). In short, we deposited the LiPON thin films both on Si wafer pieces (Okmetic Oy) and on the LHAR test structures (PillarHall and Chipmetrics Ltd.) using a commercial flow-type hot-wall ALD reactor (F-120; ASM Microchemistry Ltd.). The lithium-containing precursors LiO^*t*^Bu and Li-HMDS were chosen for this comparative conformality study because they are the two precursors so far used for LiPON ALD processes. For the source of O, P, and N, DEPA was chosen because it already contains the important P–N bond;^14^ here it should be noted that a sufficiently high N content in LiPON is crucial for its high ionic conductivity.^6^ The three precursor powders LiO^*t*^Bu, Li-HMDS, and DEPA were kept inside the reactor in open glass boats heated (unless otherwise stated) to 130, 60, and 85 °C, respectively. Nitrogen gas was generated from air and used as both the carrier and purging gas. Because the Li-HMDS + DEPA process parameters had already been optimized under similar conditions in our previous works,^8,14^ we started the present work by investigating the LiO^*t*^Bu + DEPA process in more detail. The process parameters were optimized by using Si substrates; in these experiments, the LiO^*t*^Bu precursor heating temperature, the pulse and purge lengths of both precursors, and the film deposition temperature (*T*_dep_) were varied. To confirm the linearity of the film growth, the number of ALD cycles applied was also varied.

After a few initial tests, the LiO^*t*^Bu precursor heating temperature was set to 130 °C to avoid any unwanted precursor decomposition during extended exposures to elevated temperatures.^32^ This was also the motivation behind choosing a shorter pulse length for LiO^*t*^Bu to deposit LiPON thin films with better time and material efficiency. In line with previous observations,^14^ for DEPA a relatively short pulsing time was found to be sufficient to reach surface saturation, and 3 s pulses were deemed long enough. However, LiO^*t*^Bu required comparatively longer pulse lengths (∼30 s) for full saturation (Figure 2), exhibiting a so-called soft-saturation behavior.^33^ This could be due to continued diffusion of the Li precursor material into the growing film^34,35^ or due to slower reactivity of the precursor after being briefly handled in air.^32^ Still, even when choosing a lower, not-fully saturated LiO^*t*^Bu pulse length, reasonable ALD-like growth was achieved. A temporary plateau was observed at a ∼5 s pulse length, and so it was chosen for the present study.

**Figure 2:**
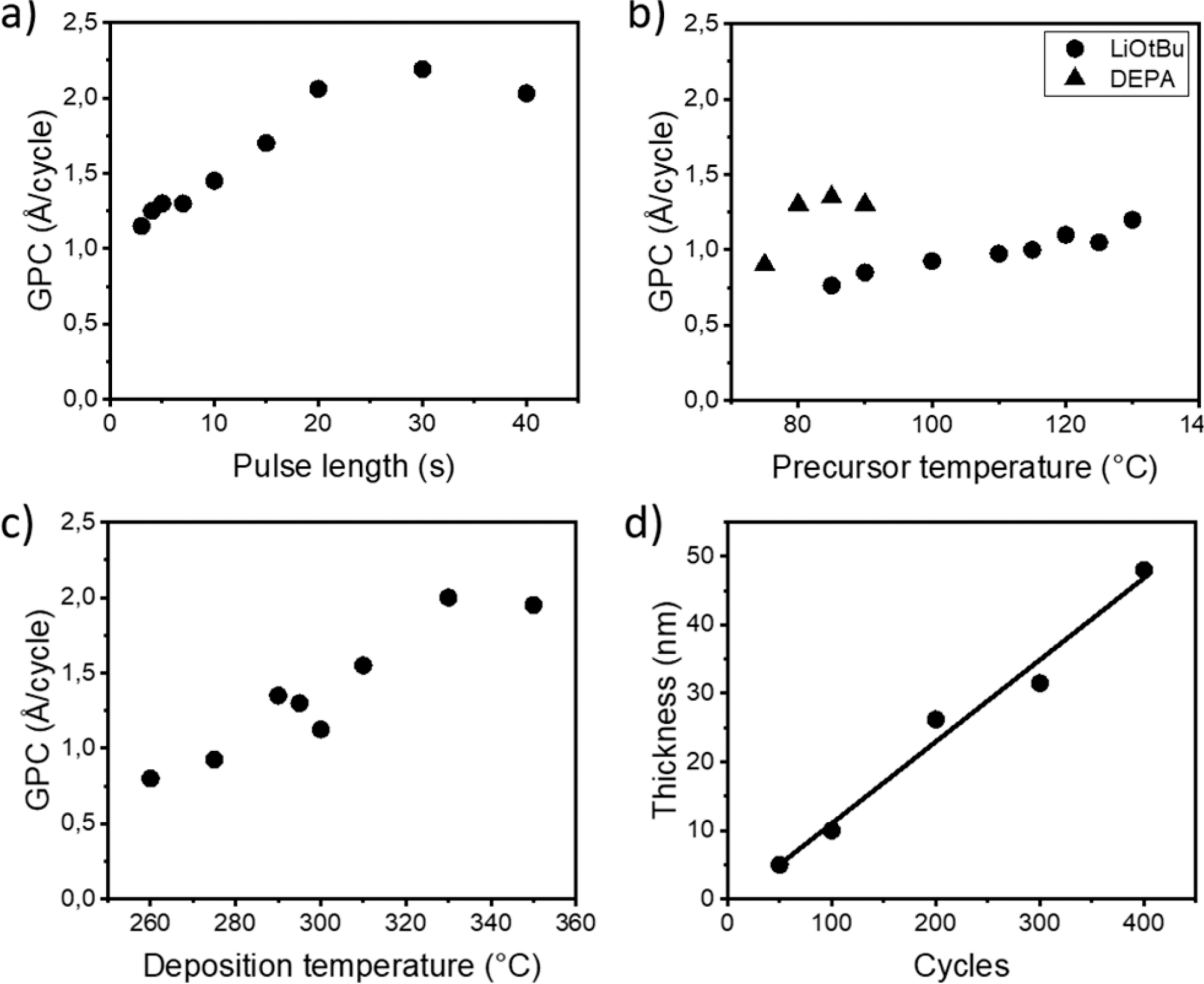
Film growth characteristics for the LiO^*t*^Bu + DEPA process: (a) LiO^*t*^Bu pulse-length saturation, (b) precursor temperature, and (c) *T*_dep_ optimizations, as well as (d) film-growth linearity confirmation. The nonvariable parameters are as listed in the Experimental Details in the SI.

For *T*_dep_ optimization, a temperature range of 260–350 °C was initially investigated for the LiO^*t*^Bu + DEPA process; an increase in the growth-per-cycle (GPC) value was seen with increasing *T*_dep_. This is somewhat different from the behavior seen for the Li-HMDS + DEPA process, for which a temperature region of constant GPC was seen between 270 and 310 °C.^14^ This difference could be due to slow saturation of the LiO^*t*^Bu precursor, where a higher temperature could aid the saturation. Another possible explanation could be a partial decomposition of precursors, as often seen for ALD processes at higher *T*_dep_ values. On the other hand, in previous literature, it had been reported for the LiO^*t*^Bu + DEPA process that the higher *T*_dep_ (in the temperature range of 250–300 °C), the lower the C impurity content in the thin film.^17^ Hence, for the rest of the experiments, we selected an intermediate *T*_dep_ of 290 °C such that it was possible to simultaneously minimize the unwanted C traces from the precursors and avoid any substantial precursor decomposition possibly occurring at *T*_dep_ values exceeding 300 °C.

Under the optimized deposition parameters at 290 °C, the LiO^*t*^Bu + DEPA process yielded visually homogeneous LiPON films on the Si substrate with GPC = 1.3 Å/cycle. For the Li-HMDS + DEPA process, the chosen parameters for the film growth at 290 °C were 3 s pulses and purges for Li-HMDS (heated at 60 °C) and 3 s pulses and purges for DEPA (heated at 85 °C), based on our previous works,^8,14^ and the resultant GPC value was 0.53 Å/cycle. We tentatively attribute the difference in the GPC values between the two Li precursors to the differences in steric hindrance because the organic ligand is significantly smaller in LiO^*t*^Bu (compared to Li-HMDS), thus explaining the higher film growth rate in the LiO^*t*^Bu + DEPA process.

The chemical bonding scheme in the films was verified by Fourier transform infrared spectroscopy (FTIR); representative spectra are displayed in Figure 3, and the interpretations of the features in the spectra are presented in Table 2. Films deposited with LiO^*t*^Bu and Li-HMDS exhibited largely similar FTIR features, particularly the characteristic wide peaks centered at ∼1030 cm^–1^ for the P–O and P–N bonds and at ∼500 cm^–1^ for the Li–O–P bonds. These results also correspond to prior ones obtained using the same equipment.^14^

**Table 2:** Interpretation of the FTIR Peaks Seen for the LiPON Films

LiO^*t*^Bu-Based LiPON		Li-HMDS-Based LiPON	
cm^–1^	contribution	cm^–1^	contribution
470–580	Li–O–P^14,36,37^	470–580	Li–O–P^14,36,37^
838	P–N=P^38^	838	P–N=P^38^
1024	P–O, P–N^14,36^	1037	P–O, P–N^14,36^
1421	CO_3_^39^	1129	P–N^36,40^
1470	CH_2_ deform^39^		
1529	N–H^38^		
1550	N–O stretch^41^		

**Figure 3:**
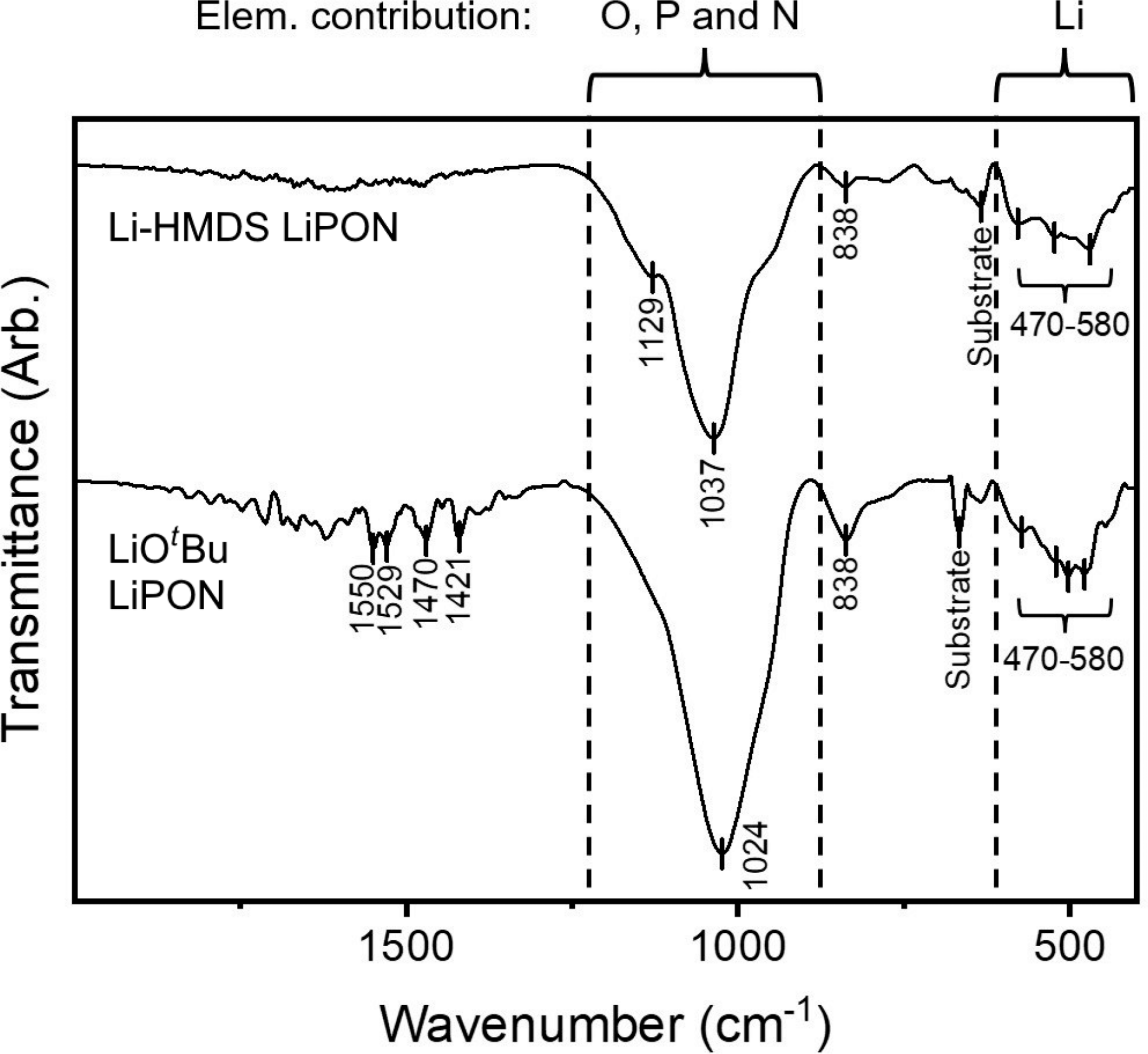
FTIR spectra for LiPON films deposited through the (top) Li-HMDS + DEPA and (bottom) LiO^*t*^Bu + DEPA processes.

After establishing the optimal deposition parameters for the two LiPON processes, we investigated the conformality, or lack thereof, of the resultant films. This was done primarily by using LHAR structures with lateral cavities corresponding to AR values varying from 10000:1 to 2:1 (Figure 4). After thin-film deposition, the top-roof poly-Si membrane was peeled off using adhesion tape, thus exposing the deposited film for straightforward conformality analysis.^30^ In the study presented here, test structures with two different gap heights (*H* = 87 and 420 nm) were used. Typically, the penetration depth (PD) of films deposited on these structures is investigated by using optical microscopy. The possibility of using optical microscopy for visualization naturally depends on the targeted film thickness and also on the intrinsic optical properties of the thin-film material itself. Accordingly, some of the LiPON films studied here were not visible under an optical microscope despite being of similar thickness to other materials successfully observed in these circumstances; this could simply be due to the optical properties of LiPON. Hence, we also utilized scanning electron microscopy (SEM) to better discern the maximum extent of film growth into the structures during the ALD process, however thin it may be.

**Figure 4:**
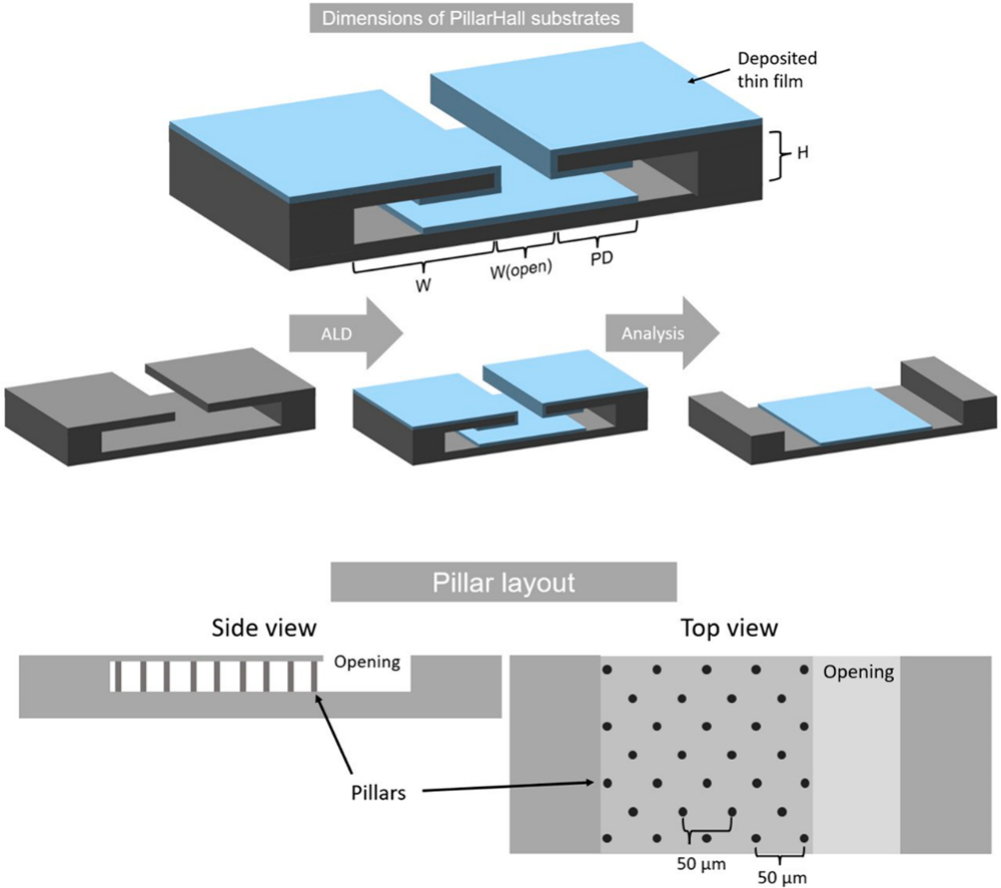
Schematics of the LHAR (PillarHall) substrates consisting of bottom and top layers connected with an array of Si micropillars. The horizontal distance between the pillars is always 50 μm, which serves as a scalebar when observing the film PD. Other important dimensions are the size of the opening area (*W*_open_) through which the precursors penetrate into the lateral cavities, the gap height (*H*) of the lateral cavities, and the cavity width (*W*). For the substrates used, *H* was either 87 or 420 nm. The *H* = 420 nm chips have multiple (both mirrored and nonmirrored) lateral cavities with *W*_open_ varying from 5 to 100 μm. The growing film is indicated in light blue; after film deposition, the top layer is peeled off such that film growth on the bottom layer can be observed/analyzed. The remains of the pillars are observed as dots in the bottom-right optical microscopy image.

First, LHAR test substrates with a 87 nm gap height were used for depositions for investigating the effect of the Li precursor choice and the precursor exposure and purging times on the PD (Figure 5). LiO^*t*^Bu was tested at pulse times of 3–7 s and compared to Li-HMDS at its previously established^14^ optimal pulse length of 3 s. Li-HMDS was also deposited at 6 s to confirm that the shorter pulse length was sufficient. To deposit ∼10 nm films with the LiO^*t*^Bu process and ∼40 nm films with both processes, 100 and 400 cycles of LiO^*t*^Bu and 600 cycles of Li-HMDS were deposited. For this comparison, we used a so-called dimensionless equivalent aspect ratio, EAR = PD/2*H*, which has been used in literature to compare the PD values in different 3D geometries.^27^ Significantly deeper penetration was observed for the LiO^*t*^Bu-based process: the visible film growth reached up to EAR = 316 and 98 for the LiO^*t*^Bu- and Li-HMDS-based processes, respectively (Figure 5).

**Figure 5:**
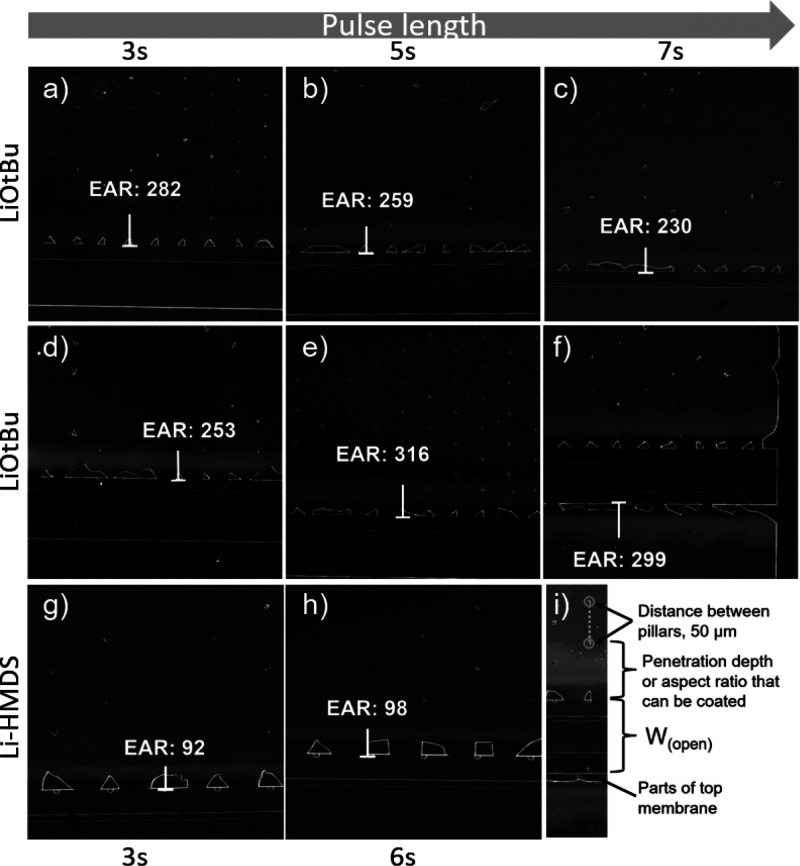
SEM images and calculated EAR values for different LiPON coatings on *H* = 87 nm LHAR chips. The data are for the two different Li precursors, (a–f) LiO*^t^*Bu and (g and h) Li-HMDS, with different precursor pulse lengths, as indicated in the figure (in each case, purge length = precursor pulse length). The cycle numbers/target film thicknesses were (a–c) 100 cycles/10 nm, (d–f) 400 cycles/40 nm, and (g and h) 600 cycles/45 nm. (i) Scale: 50 μm distance between pillars.

Increasing the pulse length should increase the precursor partial pressure inside the trenches, which helps to improve the film coverage. However, in our experiments, the PD value did not improve further beyond a 5 s pulse length, which might be caused by a further increase in the film thickness that impedes further penetration or by the diffusion limit. In previous research,^42−44^ lower GPC values have resulted in higher penetration into the lateral cavities. We suspect that the slightly increased growth rates at longer pulse lengths could be the reason behind the observed reduction in the PD because an increase in the film thickness reduces the gap height of the trench and thereby decreases the PD value.^42^

Even though an increase in the PD value was observed for the Li-HMDS + DEPA process with an increase in the Li-HMDS pulse length, the effect was not very prominent. More significantly, the different growth behaviors with the two precursors Li-HMDS and LiO^*t*^Bu indicated that Li-HMDS may be preferable when strict conformality is desired (Figure S1), while LiO^*t*^Bu can be better when aimed at extremely thin LiPON coatings that can penetrate deep into high-aspect-ratio structures (Figure 5). Indeed, according to the SEM images, while the LiPON films deposited from LiO^*t*^Bu penetrated significantly deeper in comparison to the films deposited from Li-HMDS, the latter visually appeared to have an initial region of constant growth that the LiO^*t*^Bu-based ones did not have (Figure S1). These differences can tentatively be attributed to Li-HMDS having a higher sticking coefficient.^27,45^

Because the LiO^*t*^Bu-based LiPON process seemed to allow the film to penetrate deeper into the LHAR structures, we investigated this process further by performing an additional deposition of ∼60 nm nominal thickness on a test structure with a gap height of 420 nm. In this work, nominal thicknesses for films on the LHAR structures were determined by X-ray reflectivity from a planar Si wafer set next to the LHAR chip during deposition. The wider gap height apparently increases the precursor availability inside the trenches, thus also increasing the PD value; here, the PD value determined was 299 μm in both directions of the mirrored cavity, corresponding to an EAR value of 356 based on the SEM observation (Figure 6). A similar EAR value was observed for a nonmirrored cavity, as would be expected of a conformal process (Figure S2). The same sample was also studied under an optical microscope, where the visible range of growth only extended to 50 μm depth, corresponding to EAR = 60. This image was further scrutinized via depth profiling analysis (Figure S3), which allowed us to follow the decay of the film growth until 50 μm depth; it showed an immediate and linear decline in film thickness as it entered the cavity. The deep but thin film penetration indicated by the SEM and optical data is typical of processes involving precursors with a lower sticking coefficient.^46^ In addition, ∼40 nm films were deposited on *H* = 87 nm chips, reaching a PD of 55 μm. In both cases, the LiO^*t*^Bu and DEPA pulses/purges were 5 s/5 s and 3 s/3 s, respectively.

**Figure 6:**
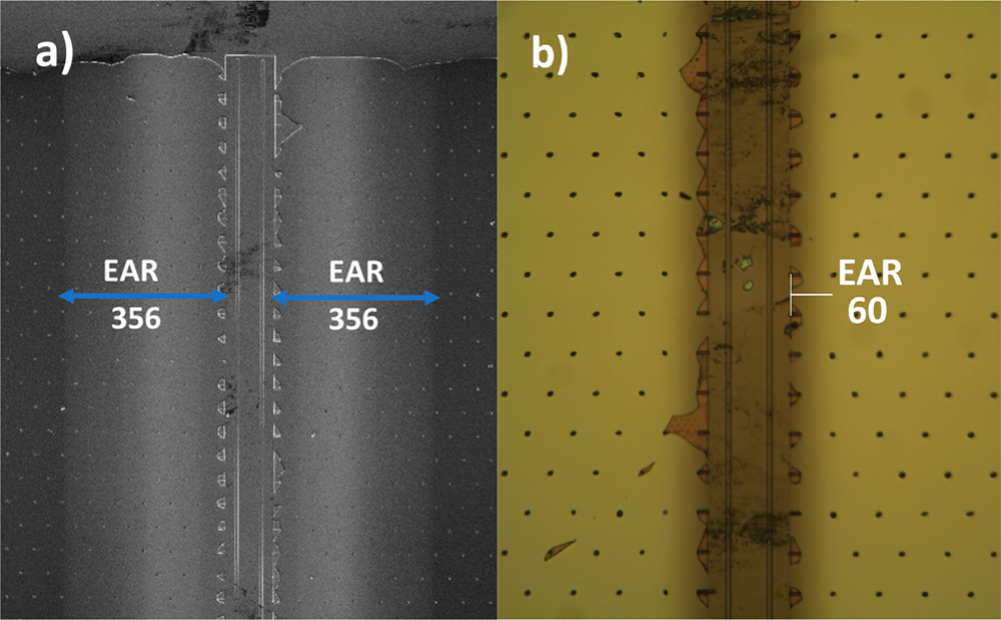
Conformality data for LiPON films grown from the LiO^*t*^Bu + DEPA process (∼60 nm nominal thickness) on a dual-sided *H* = 420 nm LHAR chip: (a) SEM image showing EAR = 356 (observed PD = 299 μm); (b) optical microscopy image showing EAR = 60 (PD = 50 μm taken where the dark area fades out, corresponding to the first contrast-change region in the SEM image). The PD values were determined by using the 50 μm horizontal distance between two pillars in the test structure.

To further examine the evenness of the deposition, another LiPON film (of 65 nm nominal thickness) was deposited on a *H* = 420 nm LHAR structure and additionally evaluated through reflectometer-based line-scan film PD profiling (Figure 7). The analysis was performed in the wavelength range of 201–1666 nm, and the refractive index remained constant at 1.6399^47,48^ throughout the wavelength range scanned, while the extinction coefficient was below 1 × 10^–5^ (Figure S4). The PD trend observed was very similar between the line scan and optical microscopy, although the PD50% values and total coverage were slightly different. The observed discrepancy may be partly explained by a hindrance in visibility through optical microscopy caused by the optical properties of the LiPON film. Together, the methods indicate that the film thickness decays along the cavity quickly in a manner consistent with the previous 60-nm-thick LiPON film. Most interestingly, the PD obtained with the line-scan reflectometry method is in accordance with the brighter portion in the SEM image (Figure 6), corresponding to a coverage of ∼160 μm, indicating how precisely the contrast difference in SEM could correlate with the thickness changes in the film. Beyond this point, the films appear ultrathin—too thin to be precisely determined by the reflectometer but still visible in SEM as another difference in contrast, likely due to the sensitivity of the method to even minute amounts of deposit.

**Figure 7:**
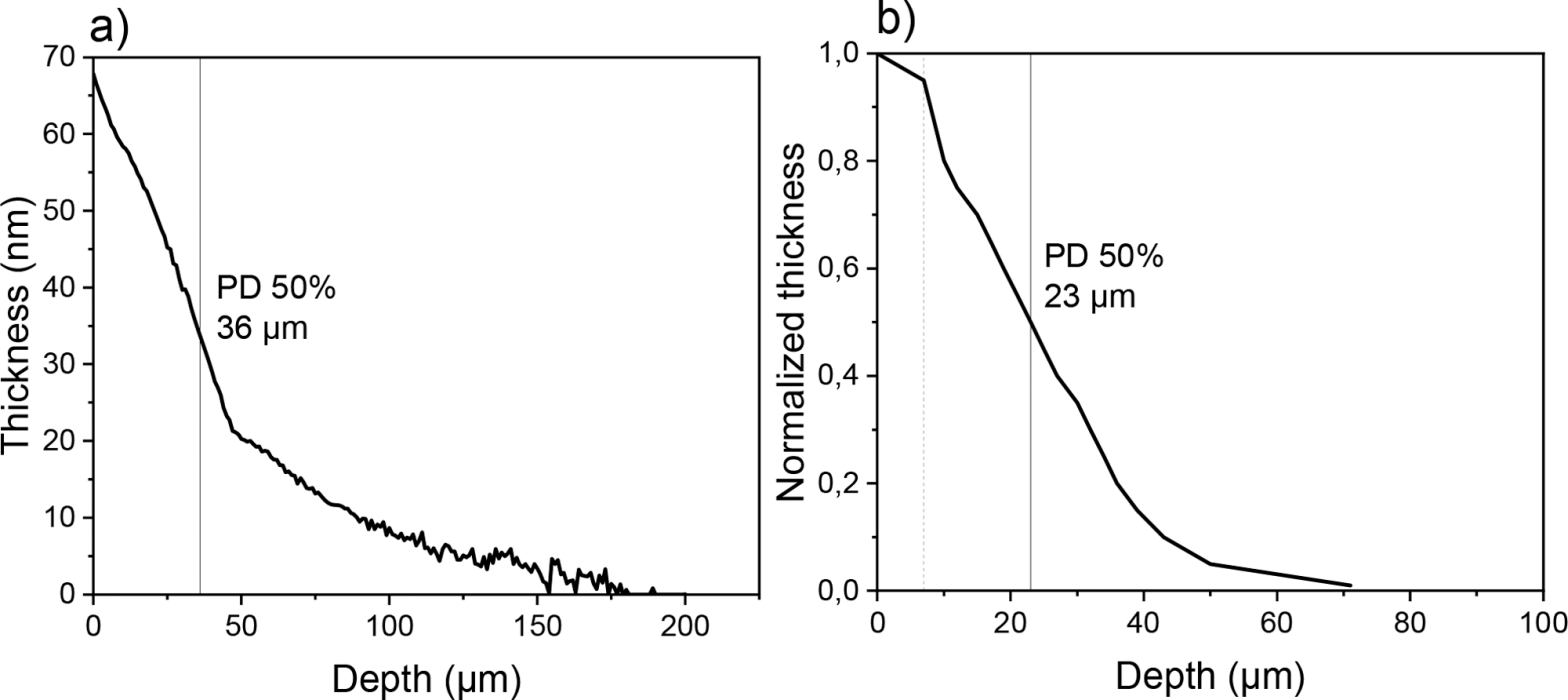
PD profiling for LiPON films grown on *H* = 420 nm LHAR chips from the LiO^*t*^Bu + DEPA process, with observed PD50% values marked for the two methods used: (a) line-scan reflectometry; (b) thickness profile determined from optical microscopy. The profile was estimated until 7 μm depth (dashed gray line) due to top membrane residue interference. The normalized thickness of 1.0 corresponds to ∼65 nm (nominal thickness).

Finally, for an additional means of testing the conformality, we performed a more rudimentary test using a previously devised^49^ simple Si sandwich structure with ∼8 μm Al foil in between to create a microscale 3D substrate with an AR value reaching up to 1250. It could be visually observed that the LiPON film grew deep into these samples, with the film thickness eventually decreasing with increasing depth (Figure S5). Thus, this simple test also verifies the significant PD of ALD LiPON films in a quantitative way; previously, some microscale 3D structures with lower AR^14,17,24^ have been used, where the deposited LiPON layer not only evenly coated the structure but even followed its nanoscale surface waviness.^14^

In conclusion, in this work, a binary thermal ALD process was developed for LiPON thin films using LiO^*t*^Bu as the lithium precursor. This process was investigated, with a focus on how evenly the films deposit onto challenging 3D surfaces. Compared to an earlier LiPON process that uses Li-HMDS as the Li source, the LiO^*t*^Bu-based process exhibited further growth in the form of deeper penetration into the LHAR test structures. A LiPON film of ∼60 nm nominal thickness grown from LiO^*t*^Bu was seen to thinly penetrate to 299 μm into a structure with only a 420 nm gap height. Even with smaller 87 nm test structures, a film of similar scale was able to extend to 50–75 μm depth. These results highlight the ability of ALD to reach a workable evenness of deposition, even with less conventional thin-film materials. Together with the technique excelling at pinhole-free films of desired thicknesses, this opens the door for studying the conformality of a large variety of battery materials deposited as advanced 3D-structured electrode coatings or as TFBs with improved power density and capacity. To further this end, future studies could be conducted by investigating LiPON films deposited onto 3D battery interfaces as well as the possible stoichiometries of LiPON achievable with different ALD process parameters, such as the precursor pulse lengths. The use of the LHAR test structures in this study provided an avenue for reliable comparative conformality studies for deposited ultrathin films, and such tools can undoubtedly be of help in similar future endeavors.

## Supporting Information Available

Experimental procedures, additional testing for LiPON ALD including SEM and optical microscopy observations for deposition on *H* = 87 nm LHAR structures (Figure S1), SEM image of LiPON on a *H* = 420 nm LHAR structure (Figure S2), thickness profile (optically analyzed) for deposition on a *H* = 420 nm LHAR structure (Figure S3), *n* and *k* values for line-scan reflectometry on a *H* = 420 nm LHAR structure (Figure S4), and simple conformality testing using a rudimentary 3D structure with an ∼8 μm gap (Figure S5) (PDF)
