# Supplementary material for: Conformal High-Aspect-Ratio Solid Electrolyte Thin Films for Li-Ion Batteries by Atomic Layer Deposition
Source: ACS Appl Electron Mater. 2024 Mar 8;6(3):1574–80. doi: 10.1021/acsaelm.3c01565 (PMC10976887; doi:10.1021/acsaelm.3c01565)
Supplement: Supplementary file 1 — el3c01565_si_001.pdf [file el3c01565_si_001.pdf]

Supporting Information

Conformal High-Aspect-Ratio Solid Electrolyte Thin Films for Li-Ion Batteries by Atomic Layer Deposition

Milad Madadi[a§], Mari Heikkinen[a§], Anish Philip[a,b] and Maarit Karppinen[a*]

[a] Department of Chemistry and Materials Science, Aalto University, FI-00076 Espoo, Finland
[b] Chipmetrics Ltd, Yliopistokatu 7, Joensuu, 80130, Finland

§ M.M. and M.H. contributed equally to this paper
*Corresponding author: maarit.karppinen@aalto.fi

EXPERIMENTAL DETAILS

Thin-film depositions: The LiPON films were deposited both on silicon wafer pieces (*Okmetic Oy*) and on *PillarHall*® lateral high-aspect-ratio (LHAR) test structures (*Chipmetrics Ltd.*) using a commercial flow-type hot-wall F-120 ALD reactor (*ASM Microchemistry Ltd.*). The precursors were lithium tert-butoxide (LiO^{t}Bu, *Alfa Aesar*, 99%), lithium bis(trimethylsilyl)amide (Li-HMDS, *SigmaAldrich*, 97%) and diethyl phosphoramidate (DEPA, *TCI Chemicals*, >98%). Unless otherwise stated, the three precursor powders, LiO^{t}Bu, Li-HMDS and DEPA, were kept inside the reactor in open glass boats heated to 130, 60 and 85 °C, respectively. Nitrogen gas (99.999%) was generated from air (*Parker HPN 5000*) and used as both carrier and purging gas. The internal pressure of the reactor was <5 mbar. Initially the deposition temperature and the precursor pulse and purge times were varied. For the final depositions, the deposition temperature was fixed to 290 °C for both processes. As the present materials were highly sensitive to humidity, extra care was taken to handle the precursors under Ar or N_2, and to store the thin-film samples in a desiccator whenever not under measurement.

Basic characterization: X-ray reflection (XRR) and grazing-incidence diffraction (GIXRD) patterns were collected (*PANanalytical X'Pert Pro*; Cu Kα1; λ = 1.540598 Å) for the films grown on Si substrates for the film thickness determination, and to confirm the lack of crystallinity. The chemical (bonding) state of the films on planar substrates was studied by Fourier-transform infrared spectroscopy (FTIR; *Bruker ALPHA II* Transmittance Spectrometer) within a wavenumber range of 400 to 4000 cm^{-1}, at a resolution of 4 cm^{-1}, and in transmission mode (32 scans). Uncoated pieces of each Si wafer were measured to subtract their absorbance from the thin-film samples spectra.

Conformality characterization: For analysing the LHAR *PillarHall*® test chips after the thin-film deposition, the polysilicon roof membrane was removed via adhesion tape approach prior to the imaging of the LiPON film grown inside the cavities. It should be emphasized that the 3D structure of these chips and the removable top membrane allow for the simple planar-like observation of cross-section-like surfaces. For the LiPON films especially, this is an important benefit, as reliable cross-sectional analysis would be highly challenging due to the instability of the LiPON material in air and the time taken to perform the analysis.

Both optical microscopy (OM; *Olympus BH-2*) and scanning electron microscopy (SEM; *Tescan Mira3* and *Zeiss VP Sigma*, 5–10 kV) imaging were used to study the film growth inside the cavities. The results are expressed using the so-called equivalent aspect ratio (EAR) value. For calculating the EAR value, the actual gap height (H) of the PillarHall chip used was measured using thin film analyser reflectometer with scanning XY-stage (*Filmetrics F40-UVX*): the actual H values were 87 and 420 nm for the chips with nominal H values of 100 and 500 nm, respectively. The same F40 UVX line-

scanning reflectometer was used for obtaining the film penetration depth profile. The measurement was indirect and is based on modelling the reflectance spectrum that best fits the measured film thickness. The complex refraction index *n* and the extinction coefficient *k* were not readily available and were determined using prior knowledge of the film thickness in the opening area of the *PillarHall* structure. Empirically, the Drude model was found to give a good estimation for *n* and *k*. The determined refraction index was used to fit the film thickness for each measured locations along the penetration depth profile.

RESULTS

Demonstration of the differences in SEM and optical microscope images: The difference between optical microscope images and SEM images with both LiPON processes is demonstrated in Figure S1. The LiO^{t}Bu-based process resulted in 55 µm penetration depth (PD) being observed by SEM (Fig. S1 a) and only 8 µm PD visible by optical microscope (Fig. S1 d) – 47 µm less than by SEM. For Li-HMDS-based LiPON, the PD difference between SEM (Fig. S1 b) and optical microscope images (Fig. S1 e) was only 2 µm.

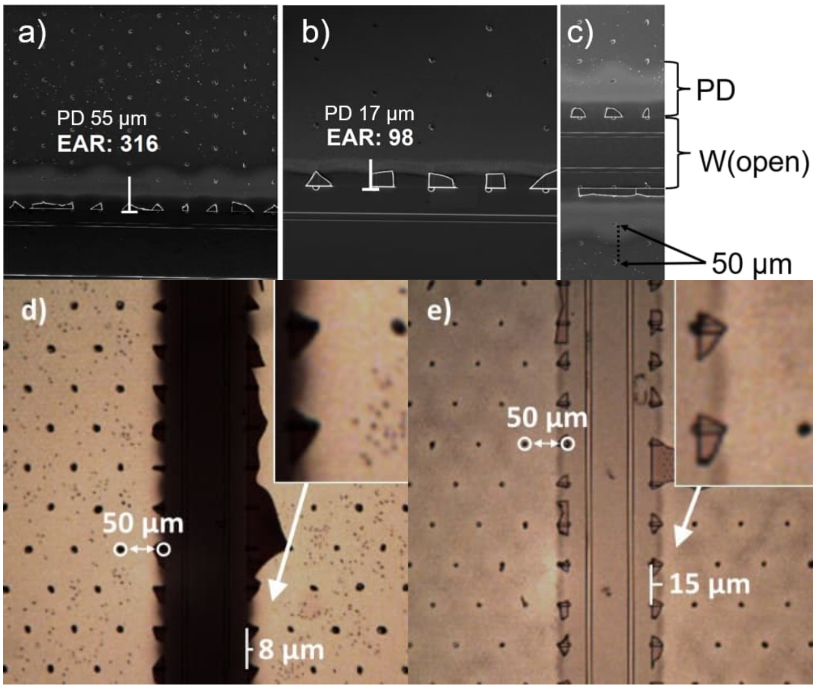

Figure S1. Microscopy observations for LiPON depositions (nominal film thickness 40 nm) within *PillarHall*® test structures with gap height H = 87 nm; the visible penetration depth (PD) is taken from the start of the cavity.

SEM images for depositions from (a) LiO^{t}Bu + DEPA process (pulse / purge times of 5 s / 5 s and 3 s / 3 s) with PD of 55 µm, and (b) Li-HMDS + DEPA process (pulse/purge times of 6 s / 6 s) with PD of 17 µm.

Schematics of (c) the test structure showing the 50 µm distance between the pillars supporting the top membrane.

Optical microscopy images for the above-mentioned samples: (d) LiO^{t}Bu-based sample with visible PD of 8 µm, and (e) Li-HMDS-based sample with visible PD of 15 µm. The inserts in (d) and (e) show a magnified view of the film coverage.

Demonstration of the penetration into non-mirrored cavity: The test structures used have both mirrored and non-mirrored lateral cavities with varying opening widths. In Figure S2, a SEM image of the non-mirrored lateral cavity is shown to give similar results as the mirrored lateral cavities.

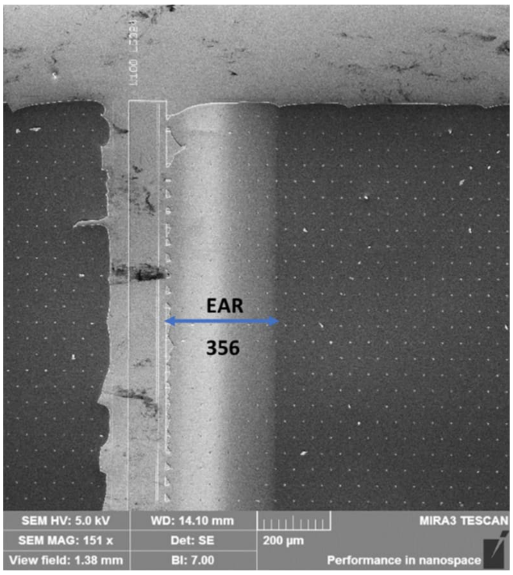

Figure S2. SEM image from a non-mirrored cavity with H = 420 nm. The EAR value calculated using the observed PD is indicated. The distance between the pillars (dots on the image) is 50 µm.

Thickness profile evaluation through optical microscopy and line-scan reflectometry: Thickness profiles of thin films along LHAR substrates can be measured in several ways. The most common technique is a reflectometry line scan. Here we demonstrate the thickness profile evaluation based on an optical microscopy image.[1] The thickness of the thin film deposited on a planar substrate in parallel with the LHAR substrate was determined by XRR to be 58 nm. Hence, the relative thickness of 1.0 in Figure S3 corresponds to 58 nm.

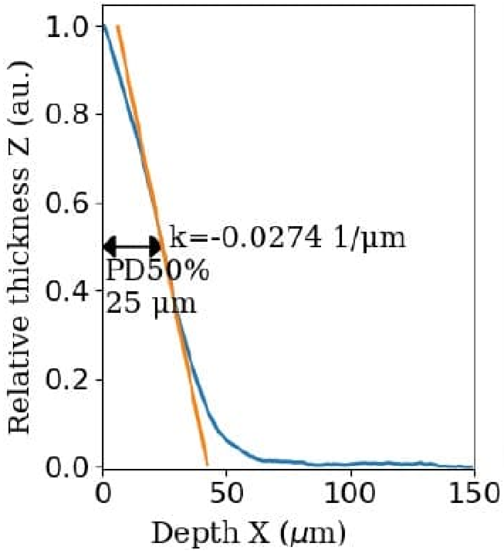

Figure S3. Thickness profile determined from an optical microscope image for a LiPON thin film grown through the LiO^{t}Bu based ALD process on H = 420 nm LHAR chip.[1] The relative thickness of 1.0 corresponds to 58 nm (measured for a similarly grown thin film on a planar substrate).

When visual data is not sufficient, line-scan reflectometry can also be utilized. Our analysis using this method is seen in Figure 7 in the main article. The refractive index *n* and extinction coefficient *k* (the parameters typically seen in spectroscopic methods) are plotted below.

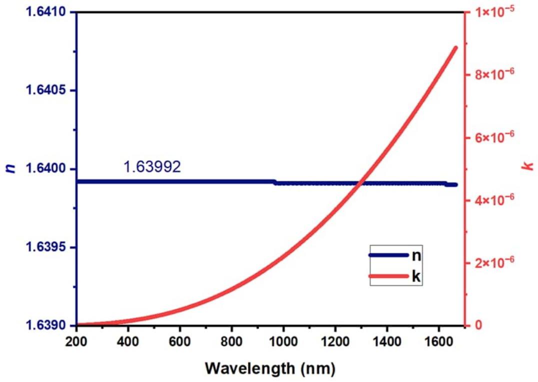

Figure S4. *n* and *k* values for line-scan reflectometry performed on a ~65 nm LiPON thin film grown using the LiO^tBu-based ALD process on H = 420 nm LHAR chip at wavelengths of 201–1666 nm.

Demonstration of the macroscale conformality characteristics of LiPON thin films grown by the two different ALD processes:

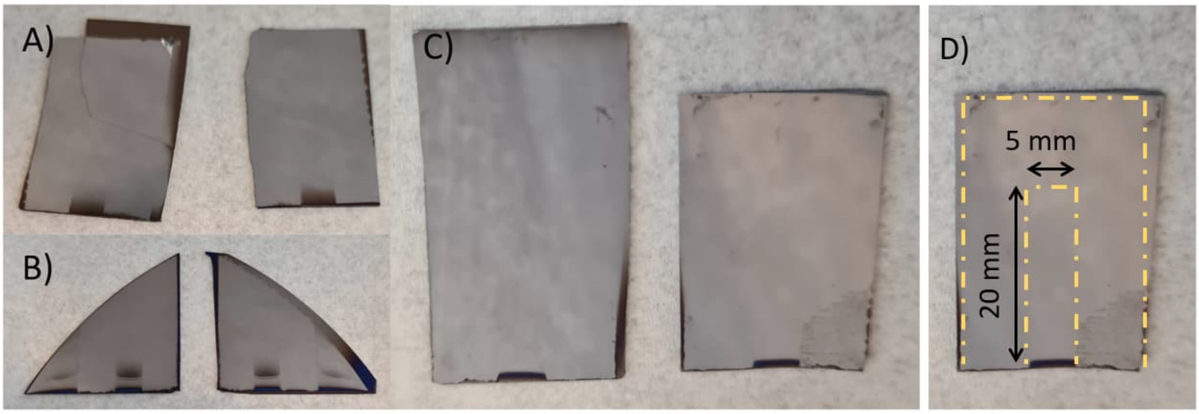

Figure S5. Conformality tests using the simple Si-wafer–Al-foil sandwich technique.[2] A) and B) Depositions with the LiO^tBu + DEPA process (pulse/purge times of 10 s/10 s). C) Depositions with the Li-HMDS + DEPA process (pulse/purge times of 3 s/3 s). In our setup, we used ~8 µm thick aluminum foil, which was then cut so that the lateral cavity was 5 mm wide and 20 mm long, as shown in D). The dimensions used in this study resulted in an AR of 1250 for the whole cavity according to the following formula: AR = $Lp/4A$, where L is the depth of the cavity, p is the perimeter of the opening and A is the area.[2]

References

(1) Utriainen, M.; Saastamoinen, K.; Rekola, H.; Ylivaara, O. M. E.; Puurunen, R. L.; Hyttinen, P. Optical Metrology of 3D Thin Film Conformality by LHAR Chip Assisted Method. *In SPIE OPTO 2022, Proceedings of SPIE 12008, Photonic Instrumentation Engineering IX*, San Francisco, United States, March 5, 2022; Buse, L. E.; Soskind, Y.; 120080D. DOI: 10.1117/12.2609643

(2) Dendooven, J.; Deduytsche, D.; Musschoot, J.; Vanmeirhaeghe, R. L.; Detavernier, C. Modeling the Conformality of Atomic Layer Deposition*:* The Effect of Sticking Probability*. J. Electrochem. Soc.* 2009, *156*, P63–P67. DOI: 10.1149/1.3072694.
